# Custom insoles versus sham and GP-led usual care in patients with plantar heel pain: results of the STAP-study - a randomised controlled trial

**DOI:** 10.1136/bjsports-2019-101409

**Published:** 2020-09-02

**Authors:** Nadine Rasenberg, Sita M A Bierma-Zeinstra, Lars Fuit, Michael Skovdal Rathleff, Amy Dieker, Peter van Veldhoven, Patrick J E Bindels, Marienke van Middelkoop

**Affiliations:** 1 Department of General Practice, Erasmus MC University Medical Center, Rotterdam, The Netherlands; 2 Podiatrist practice: Podotherapie Fuit en van Houten, Rijswijk, The Netherlands; 3 Center for General Practice in Aalborg, Aalborg University, Aalborg, Denmark; 4 Dutch Association of Podiatrists, Hilversum, The Netherlands; 5 Department of Sports Medicine, Haaglanden Medical Centre, Leidschendam, The Netherlands

**Keywords:** foot, general practice, intervention, podiatry

## Abstract

**Objectives:**

To compare custom-made insoles to sham insoles and general practice (GP)-led usual care in terms of pain at rest and during activity at 12 weeks follow-up in individuals with plantar heel pain.

**Methods:**

In this randomised clinical trial 185 patients aged 18 to 65 years, with a clinical diagnosis of plantar heel pain for at least 2 weeks, but no longer than 2 years were recruited. Patients were randomly allocated into three groups: (1) GP-led treatment, plus an information booklet with exercises (usual care; n=46), (2) referral to a podiatrist for treatment with a custom-made insole plus an information booklet with exercises (custom-made insole; n=70) and (3) referral to a podiatrist and treatment with a sham insole plus an information booklet with exercises (sham insole; n=69). As well as the primary outcome of pain severity (11-point Numerical Rating Scale) we used the Foot Function Index (0 to 100) as a secondary outcome.

**Results:**

Of 185 randomised participants, 176 completed the 12-week follow-up. There was no difference in pain or function between the insole and the sham groups at 12 weeks. Participants in the GP-led usual care group reported less pain during activity at 12 weeks, (mean difference (MD) 0.94, 95% CI 0.23 to 1.65), less first step pain (MD 1.48, 95% CI 0.65 to 2.31), better function (MD 7.37, 95% CI 1.27 to 13.46) and higher recovery rates (RR 0.48, 95% CI 0.24 to 0.96) compared with participants in the custom insole group.

**Conclusions:**

Referral to a podiatrist for a custom-made insole does not lead to a better outcome compared to sham insoles or compared to GP-led usual care.

**Trial registration number:**

NTR5346.

## Introduction

Plantar heel pain, also known as plantar fasciitis or fasciopathy, is a common cause of foot pain. Plantar heel pain accounts for an estimated 8% to 15% of all foot complaints requiring medical care in adults.^1 2^ Plantar heel pain typically affects highly physically active people, such as running athletes, but is also common in middle-aged (40 to 60 years) women with high body mass index (BMI).^3–6^ The incidence of plantar heel pain in Dutch general practice is approximately 3.8 per 1000 patient-years.^7^ The clinical course of plantar heel pain is considered favourable with remission after 12 to 24 months in 60% to 80% of patients.^8 9^ However, given the effect of the complaints on every day and sports activities, the burden on patients is high.^10^ There is a need for effective treatment options, that can speed up recovery and limit impact.

Plantar heel pain can be managed by a range of different treatments in clinical practice and orthoses, such as in-shoe foot insoles are commonly applied by clinicians.^11^ Two systematic reviews found conflicting evidence for the effectiveness of orthoses on pain in plantar heel pain.^12 13^ Whittaker *et al* found a small, but statistically significant, beneficial effect of insoles on heel pain at 7 to 12 weeks when compared with sham.^12^ In contrast, a more recent systematic review concluded that insoles are not superior to sham in reducing pain in individuals with plantar heel pain.^13^ Of the three trials comparing custom-made insoles to sham, none were performed in a primary care setting.^13^ Additionally, no study has compared orthoses to usual care, despite custom-made insoles being the most frequent applied interventions for plantar heel pain in general practice.^7^ The added effect of insoles to sham is unclear and to usual care is unknown, making it impossible to know if referral for custom-made insoles is an appropriate option for patients who consult general practice.

We conducted a randomised clinical trial with a pragmatic design that compared custom-made insoles with sham insoles, as well as general practice (GP)-led usual care. The hypothesis was that custom-made insoles would be better than sham insoles and GP-led usual care at 12 weeks follow-up in terms of pain at rest and during activity.

## Methods

### Trial design

The study was performed according to the published protocol.^14^ Briefly, we performed a pragmatic three-armed participant-blinded and assessor-blinded randomised controlled trial in primary care, where usual care by the GP or sports physician was compared with referral to a podiatrist for a custom-made insole and to referral to a podiatrist for a sham insole, with a follow-up of 6 months. Participants provided informed consent.

### Participants

A total of 175 GPs and 6 sports physicians were engaged in the study and invited patients with plantar heel pain to participate in the study. Inclusion criteria for study participation were: age between 18 to 65 years, minimal pain duration of 2 weeks and presentation with plantar heel pain, characterised as pain at the medial hind foot. Exclusion criteria were: recurrent complaints of plantar heel pain for more than 2 years, complaints caused by trauma, earlier treatment for plantar heel pain by a podiatrist or with insoles, suspected (by the GP or sports physician) osteoarthritis in the subtalar or talonavicular joint, suspected tarsal tunnel syndrome, suspected stress fractures, infections or tumours in the painful foot, presence of systemic diseases (such as ankylosing spondylitis, psoriasis or multiple sclerosis) and insufficient understanding of the Dutch language. Patients who were regarded eligible by the GP or sports physician and were interested in the study were double screened on eligibility by the research assistant by telephone.

#### Randomisation and blinding

Participants were randomised with the use of a computer-generated randomisation list using block randomisation with random block sizes between 3 and 10 with a 2:3:3 allocation randomisation ratio, to receive the following interventions:

- Referral to podiatrist for custom-made insole (custom-made insole).
- Referral to podiatrist for sham insole (sham).
- GP-led usual care (usual care).

The randomisation list was created by an independent person (data manager) and the sequence was hidden from all involved researchers. Randomisation was stratified for type of referral (GP or sports physician). Patients were blinded to the type of insole they received. Podiatrists were blinded during the first consultation, but received information necessary to fabricate the insole after the first consultation and were no longer blinded afterwards. Podiatrists were instructed to not inform the patients about the allocation during the course of the study. GPs remained blinded as well as they did not receive any information on group allocation.

### Interventions

Participants allocated to the usual care group received usual care by their GP or sports physician. This included a non-surgical approach and any intervention the physician considered to be necessary for each particular patient, except a referral to a podiatrist.

Participants allocated to the custom-made insole group and the sham insole group, were referred to one of the 50 participating podiatrists. In the Netherlands, a podiatrist is a certified paramedical specialist providing podiatric care. Participants referred to a podiatrist received a standardised assessment, including the making of a 3D (three-dimensional) imprint of the feet of the patient. After this intake, the podiatrist contacted the research team to receive the allocation of the patient. Consequently, participants randomised to the custom-made insole group received a custom-made insole, which was manufactured at the discretion of the individual podiatrist. Therefore, multiple approaches were used. The common goal was to influence the biomechanical process to reduce traction on the plantar aponeurosis and to reduce ground reaction force below the calcaneal tuberosity. To achieve this, most applied full-length insoles with shock absorbing material (PPT/Poron of 15 Shore A) at the calcaneus, with or without a shell shape under the attachment of the medial portion of the plantar aponeurosis. A corrective element for the calcaneus aimed to correct range of motion of the tarsus in frontal plane and often an additional arch support was applied. The material used for the insoles was of 30 to 60 Shore A. Participants randomised to the sham insole group received a sham insole that was designed for each participant (based on the 3D imprint) and designed to have the visual effect of a podiatric insole, but providing as little mechanical effect as possible. All sham insoles were produced by the same podiatrist after receiving the 3D imprint from the different podiatrists. The sham insoles were sent back to the different podiatrists to give to the patients. The detailed procedure for making the sham insole is described elsewhere.^14^ The procedures on the standardised assessment, the allocation concealment and the manufacturing of the custom and sham insole were agreed on in a consensus meeting with participating podiatrists.

Participants in all three groups received an information booklet with general information on plantar heel pain, as well as information on stretching and strengthening exercises, based on those described by Digiovanni *et al* and Rathleff *et al* 9 15

Podiatrists were instructed to provide patients with the allocated insole and to provide exercises and shoe advice when needed and to withhold other interventions available to them. Physicians were instructed not to refer patients in the usual care group to a podiatrist during the study period. Other interventions, including the prescription of paracetamol or non-steroidal anti-inflammatory drugs, as well as all other co-interventions, were left to the physician’s decision.

### Outcomes

Participants completed online questionnaires at baseline, 2, 4, 6, 12 and 26 weeks of follow-up. At baseline, information on demographics including the activity score (in tertiles) based on the SQUASH questionnaire (ShortQuestionnaire to Assess Health-enhancing Physical Activity) was gathered.^16^ The primary outcomes were the differences in pain at rest and during activity on a 11-point Numerical Rating Scale (NRS) at the 12-week follow-up between the custom insole group and sham group, and compared with usual care.^17^ Secondary outcomes included, first step pain, the Foot Function Index (FFI 0 to 100) and the self-reported recovery on a 7-point Likert scale after 12 weeks follow-up.^18 19^ Pain, recovery and foot function were measured at all time points. Quality of life according to SF12 (The12-Item Short Form Health Survey) was measured at baseline, 12 weeks and 26 weeks of follow-up.^20^ The 26-week follow-up questionnaire additionally included items on compliance, patient satisfaction and success of blinding. For participants allocated to an insole, the podiatrist reported whether they agreed with the referral based on their findings at the baseline assessment.

### Statistical analysis and sample size

Sample size was based on the ability to detect a clinically relevant effect size of 0.5, translated to a difference in pain score of 10.5 (SD 21.5, based on Landorf *et al*) on a scale of 0 to 100 between patients with a sham insole and custom-made insole at the 12-week follow-up.^21 22^ The sample size of the usual care group was based on the expected larger difference (12 points on 0 to 100 scale) between usual care and custom-made insole, as described in the protocol.^14^ Differences between the insole groups were analysed following the intention-to-treat principle. For the continuous outcomes linear mixed models with repeated measures were used to compare the intervention group and control groups and results expressed in mean differences (MD). To model the covariance of repeated measures by patients, the option for data structure in the analyses was set on ‘Unstructured’, because this had the lowest Akaike’s information criterion. Fixed effects were time and time by treatment. All time measures of the outcome taken before the outcome of interest (including baseline values) were included in the analyses. For the outcome self-reported recovery at 6, 12 and 26 weeks, generalised linear models (with a logit link and binomial distribution) were used and results expressed in relative risks (RR). Missing data were handled with restricted maximum likelihood which generates unbiassed estimates of the population covariance parameters and does not reject cases where one or more data items are missing. For self-reported recovery, the number needed to treat is presented (defined as 1/absolute relative risk) and for continuous data, effect sizes (Hedges g) with accompanied CIs were calculated.

A Bonferroni correction was performed for the secondary comparisons to the usual care group. The analyses were adjusted for potential confounders at least including age, sex, BMI and activity level and variables that changed the effect estimate of the outcome of interest by >10%. Potential confounders were tested for collinearity. Predefined subgroup analysis as described in the protocol were performed.^14^ All analyses were performed with IBM SPSS Statistics (V.25).

## Results

### Participants

**Figure 1:**
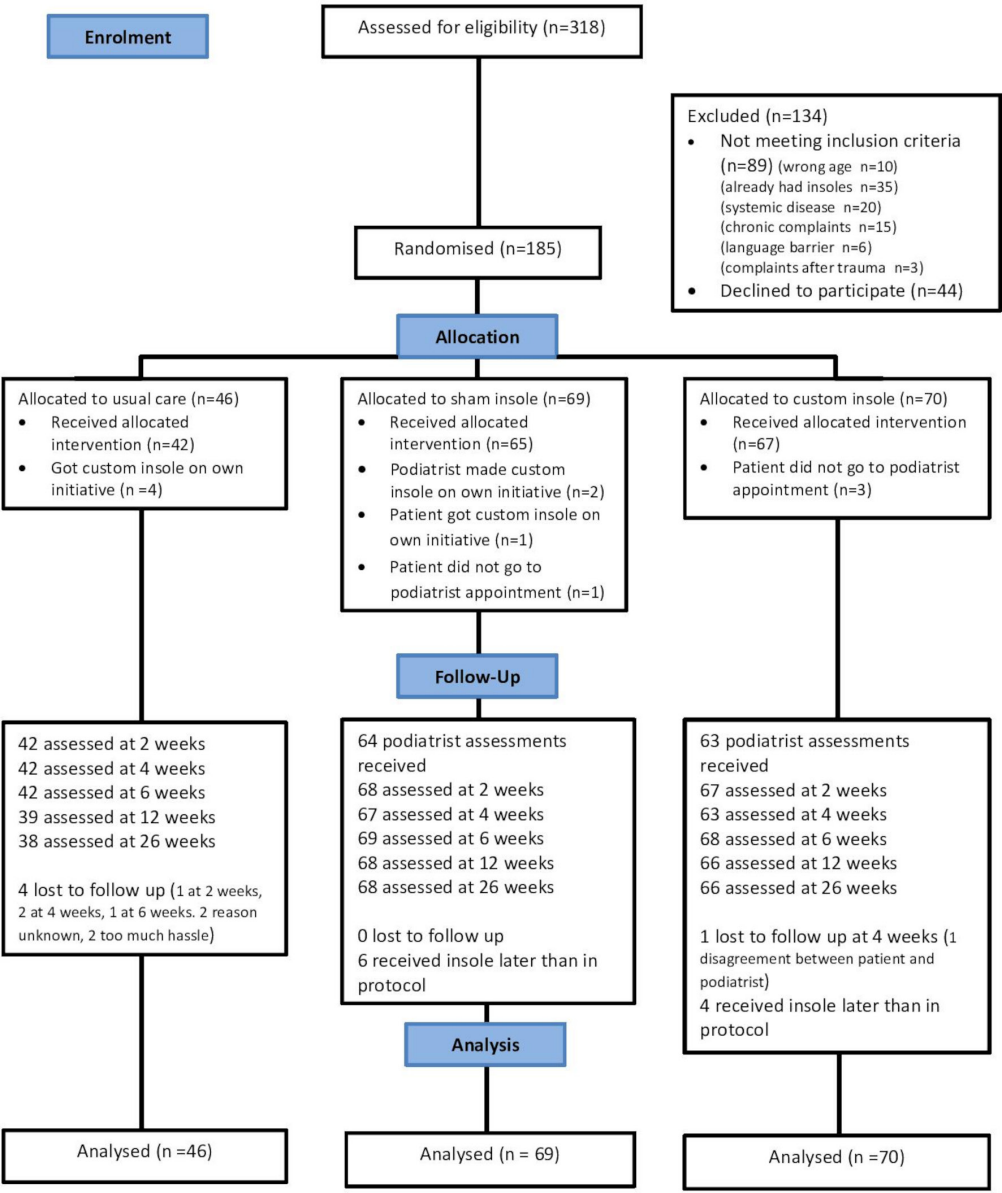
Flowchart depicting the flow of patients in the STAP-study.

Inclusion lasted from September 2015 until May 2018 with the last follow-up being conducted in November 2018. Three hundred and eighteen patients were interested in the study after being informed by their GP. Of these, 185 participants were eligible and included: 70 in the custom-made insole group, 69 in the sham insole group and 46 in the usual care group. The flow of patients is presented in figure 1. All 185 participants were referred to the study by their GP, there were no referrals by sports physicians. Baseline demographics and characteristics are reported in table 1.

**Table 1:** Baseline characteristics of the STAP-study participants (n=185)

	Total population N=185	Custom-made insole group n=70	Sham insole group n=69	Usual care group n=46
	Mean (SD) unless otherwise indicated	Mean (SD) unless otherwise indicated	Mean (SD) unless otherwise indicated	Mean (SD) unless otherwise indicated
Age, y	47.6 (10.6)	48.0 (11.3)	48.2 (9.4)	46.1 (11.4)
Sex, female, No (%)	128 (69.2)	48 (68.6)	48 (69.6)	32 (69.6)
Educational level, No (%)				
Low	59 (31.9)	23 (32.9)	20 (29.0)	16 (34.8)
Middle	86 (46.5)	29 (41.4)	33 (47.8)	24 (52.2)
High	40 (21.6)	18 (25.7)	16 (23.2)	6 (13.0)
BMI	29.7 (5.3)	29.2 (5.8)	29.5 (4.8)	30.9 (5.0)
Pain history				
Localization of complaints, bilateral, No (%)	45 (24.3)	16 (22.9)	16 (23.2)	13 (28.3)
Duration of pain, mo	6.2 (10.4)	7.7 (15.5)	5.1 (5.2)	5.4 (5.6)
VAS during rest (0–10)	4.1 (2.6)	3.8 (2.5)	4.0 (2.7)	4.9 (2.4)
VAS during activity (0–10)	6.8 (2.0)	6.8 (2.0)	6.7 (2.1)	7.0 (1.8)
VAS first step pain (0–10)	7.2 (2.3)	7.2 (2.4)	7.3 (2.1)	7.2 (2.5)
DN4 (0–10>4indicates neuropathic pain)	3.7 (2.1)	3.9 (2.1)	3.6 (1.8)	3.7 (2.3)
FFI total (0–100)	48.7 (18.0)	50.2 (18.8)	46.1 (17.2)	50.3 (18.0)
FFI disability (0–100)	39.6 (20.9)	41.6 (23.1)	37.3 (19.7)	40.1 (19.3)
FFI pain (0–100)	58.7 (17.7)	60.0 (16.7)	55.6 (17.2)	61.2 (19.6)
FPDI function (9-27)	17.5 (4.4)	17.2 (4.2)	17.6 (4.9)	17.9 (4.2)
Self-reported illness in the past 12 months, No (%)	116 (62.7)	45 (64.3)	45 (65.2)	26 (56.5)
Other musculoskeletal pain at baseline, No (%)	78 (42.2)	29 (41.4)	32 (46.4)	17 (37.0)
Quality of life				
SF12 Physical health (0–100)	38.2 (8.4)	39.0 (8.4)	37.9 (8.7)	37.4 (7.8)
SF12 Mental health (0–100)*	49.2 (10.1)	46.9 (11.1)*	51.2 (8.9)*	49.5 (9.9)
EQ-5D Utility score (0–1)	0.7 (0.2)	0.7 (0.3)	0.7 (0.2)	0.7 (0.2)
Activity level				
SQUASH	7716.7 (5270.0)	6761.3 (4525.5)	8755.3 (5747.8)	7612.6 (5398.8)
Self-reported interventions for PHP in the 3 months prior to inclusion				
Visit to GP, No (%)	165 (89.2)	60 (85.7)	62 (89.9)	43 (93.5)
Visit to physiotherapist, No (%)	15 (8.1)	6 (8.6)	5 (7.2)	4 (8.7)
Use of pain medication, No (%)	73 (39.5)	30 (42.9)	25 (36.2)	18 (39.1)
Exercises, No (%)	73 (39.5)	26 (37.1)	29 (42.0)	18 (39.1)
Shockwave, No (%)	2 (1.1)	2 (2.9)	–	–
Dry Needling, No (%)	1 (0.5)	–	1 (1.4)	–
Massage/ manipulation, No (%)	4 (2.2)	1 (1.4)	3 (4.3)	–
Custom insoles, No (%)	1 (0.5)	1 (1.4)	–	–
Prefabricated orthotic, No (%)	67 (36.2)	29 (41.4)	25 (36.2)	13 (28.3)
Shoe advice, No (%)	14 (7.6)	4 (5.7)	8 (11.6)	2 (4.3)
Corticosteroid injection, No (%)	3 (1.6)	–	2 (2.9)	1 (2.2)

*There was a mean difference of 4.28 (95% CI 0.18 to 8.38) on the mental health component of the SF12 between the sham insole group and the custom-made insole group, in favour of sham insole.

BMI, body mass index; DN4, Douleur Neuropathique 4; EQ-5D, EuroQol five dimension scale; FFI, Foot Function Index (total score and pain and disability subscales); FPDI, (Manchester) Foot Pain and Disability Index (function subscale); mo, months; No, number of participants; SF12, The 12-Item Short Form Health Survey; SQUASH, Short Questionnaire to Assess Health-enhancing Physical Activity; VAS, Visual Analogue Scale; y, years.

### Primary and secondary outcomes

No differences were seen between the custom-made insole and the sham insole group in pain and function at 12 weeks (table 2). The 95% CIs of this comparison exclude any relevant favourable effect of the custom-made insole. After 12 weeks, the group randomised to GP-led usual care showed significantly larger improvements compared with the group randomised to custom insoles in pain during activity (mean difference (MD) 0.94, 95% CI 0.23 to 1.65), first step pain (MD 1.48, 95% CI 0.65 to 2.31), FFI pain subscale (MD 6.27, 95% CI 0.84 to 11.69), FFI function subscale (7.37, 95% CI 1.27 to 13.46) and self-reported recovery (RR 0.48, 95% CI 0.24 to 0.96). The differences were small compared with the known minimal clinical differences; only the FFI function subscale exceeded the minimal clinical important difference of 7.^21 23^

**Table 2:** Intention-to-treat-analysis: results of the multivariable linear mixed model analysis with repeated measures and study group comparisons

No of weeks after start of intervention	Custom-made insole	Sham insole	Usual care	Custom-made insole versus sham insole*			Custom-made insole versus usual care†		
				MD (95% CI; P)	RR (95% CI; P)	Effect size/NNT (95% CI)	MD (95% CI; P)	RR (95% CI; P)	Effect size/NNT (95% CI)
Pain at rest (crude mean (SD) score on pain numerical rating scale (0–10); lower score indicates less pain; adjusted mean difference measured between groups‡; effect size Hedges g)									
6	3.99 (2.71)	4.32 (2.53)	3.48 (2.86)	−0.41 (−1.11 to 0.29; 0.25)		−0.15 (−0.48 to 0.19)	−0.30 (−1.13 to 0.52; 0.47)		−0.12 (−0.49 to 0.25)
12	3.29 (2.84)	3.38 (2.72)	3.21 (3.10)	−0.34 (−1.03 to 0.35; 0.34)		−0.14 (−0.48 to 0.19)	−0.16 (−0.95 to 0.64; 0.70)		−0.06 (−0.44 to 0.31)
26	2.39 (2.67)	2.49 (2.71)	2.55 (3.27)	−0.33 (−1.00 to 0.34; 0.33)		−0.17 (−0.50 to 0.16)	−0.19 (−0.98 to 0.60; 0.64)		−0.08 (−0.45 to 0.30)
Pain during activity (crude mean (SD) score on pain numerical rating scale (0–10); lower score indicates less pain; adjusted mean difference measured between groups§; effect size Hedges g)									
6	5.72 (2.62)	5.75 (2.21)	4.43 (2.87)	−0.07 (−0.61 to 0.47; 0.80)		−0.04 (−0.37 to 0.30)	**0.96 (0.25 to 1.68; 0.01**)		0.46 (0.08 to 0.84)
12	4.59 (3.00)	4.69 (2.61)	4.13 (2.61)	−0.05 (−0.58 to 0.49; 0.87)		−0.02 (−0.36 to 0.31)	**0.94 (0.23 to 1.65; 0.01**)		0.45 (0.08 to 0.83)
26	3.98 (2.93)	3.53 (3.00)	3.42 (3.55)	0.07 (−0.46 to 0.60; 0.80)		0.04 (−0.29 to 0.37)	**0.91 (0.20 to 1.62; 0.01**)		0.44 (0.07 to 0.82)
First step pain (crude mean (SD) score on pain numerical rating scale (0–10); lower score indicates less pain; adjusted mean difference measured between groups¶; effect size Hedges g)									
6	5.82 (3.03)	6.19 (2.41)	4.55 (3.30)	0.00 (−0.63 to 0.63; 1.00)		0.00 (−0.33 to 0.33)	**1.57 (0.73 to 2.40; 0.00**)		0.63 (0.25 to 1.02)
12	4.61 (3.45)	4.84 (2.94)	4.26 (3.58)	0.01 (−0.61 to 0.63; 0.98)		0.00 (−0.33 to 0.34)	**1.48 (0.65 to 2.31; 0.00**)		0.60 (0.22 to 0.98)
26	3.77 (3.51)	3.46 (3.20)	3.11 (3.30)	0.12 (−0.50 to 0.74; 0.71)		0.06 (−0.28 to 0.39)	**1.43 (0.61 to 2.26; 0.01**)		0.59 (0.21 to 0.97)
FFI-pain subscale (crude mean (SD) score on pain numerical rating scale (0–100); lower score indicates less pain; adjusted mean difference measured between groups**; effect size Hedges g)									
6	48.21 (22.57)	48.32 (20.29)	37.23 (25.67)	−1.35 (−5.60 to 2.91; 0.53)		−0.09 (−0.43 to 0.24)	**6.45 (1.10 to 11.80; 0.02**)		0.41 (0.04 to 0.79)
12	37.70 (23.82)	38.04 (22.47)	36.17 (27.52)	−1.07 (−5.38 to 3.24; 0.62)		−0.07 (−0.41 to 0.26)	**6.27 (0.84 to 11.69; 0.02**)		0.40 (0.03 to 0.78)
26	31.68 (22.28)	31.09 (27.43)	26.26 (28.55)	−0.87 (−5.41 to 3.68; 0.71)		−0.06 (−0.39 to 0.27)	**6.50 (0.84 to 12.15; 0.03**)		0.40 (0.03 to 0.78)
FFI-function subscale (crude mean (SD) score on function numerical rating scale (0–100); lower score indicates better function/less disability; adjusted mean difference measured between groups††; effect size Hedges g)									
6	33.30 (25.24)	32.77 (21.91)	26.28 (23.23)	1.12 (−4.40 to 6.34; 0.69)		0.06 (−0.27 to 0.39)	**7.46 (1.14 to 13.79; 0.02**)		0.38 (0.01 to 0.76)
12	24.73 (23.87)	23.33 (21.45)	24.82 (22.98)	1.77 (−3.69 to 7.22; 0.52)		0.09 (−0.24 to 0.43)	**7.37 (1.27 to 13.46; 0.02**)		0.39 (0.02 to 0.77)
26	18.87 (22.47)	18.87 (23.40)	18.55 (25.53)	1.89 (−3.54 to 7.32; 0.49)		0.10 (−0.23 to 0.43)	**7.07 (1.01 to 13.13; 0.02**)		0.38 (0.00 to 0.75)
FFI total (crude mean (SD) score on function numerical rating scale (0–100); lower score indicates better function/less disability; adjusted mean difference measured between groups‡‡; effect size Hedges g)									
6	40.25 (22.61)	40.09 (20.01)	31.40 (23.99)	−2.70 (−6.30 to 0.90; 0.14)		−0.24 (−0.57 to 0.09)	3.47 (−1.35 to 8.29; 0.16)		0.26 (−0.11 to 0.64)
12	30.83 (23.16)	30.29 (20.98)	30.16 (24.58)	−2.30 (−6.18 to 1.57; 0.24)		−0.19 (−0.53 to 0.14)	3.26 (−1.85 to 8.37; 0.21)		0.24 (−0.14 to 0.61)
26	24.96 (23.08)	24.61 (24.09)	22.23 (24.51)	−2.12 (−6.24 to 2.00; 0.31)		−0.17 (0.50 to 0.17)	3.31 (−2.08 to 8.69; 0.23)		0.23 (−0.15 to 0.60)
Self-reported recovery (No (%) of participants reporting recovery of their plantar heel pain symptoms (Recovery=when participants have reported ‘completely recovered’ or ‘mostly recovered’ on a 7-point Likert scale); lower score indicates less recovery; adjusted RR measured between groups; NNT)									
6	8/68 (11.4)	7/69 (10.1)	11/42 (26.2)		2.01 (0.77 to 5.28; 0.16)	61.7 (8.3 to −11.3)		**0.34 (0.14 to 0.86; 0.02**)	−6.9 (−3.4 to 108.9)
12	24/66 (36.4)	25/69 (36.2)	15/41 (36.6)		1.22 (0.68 to 2.19; 0.50)	759.0 (6.1 to −6.2)		**0.48 (0.24 to 0.96; 0.04**)	−451.0 (−5.3 to 5.4)
26	35/67 (52.2)	39/68 (57.4)	21/38 (55.3)		1.01 (0.65 to 1.57; 0.96)	−19.6 (−4.6 to 8.6)		0.61 (0.34 to 1.08; 0.09)	−33.1 (−4.4 to 6.0)
SF-12 physical health component (crude mean (SD) score on quality of life (0–100); lower score indicates less quality of life; adjusted mean difference measured between groups§§)									
12	42.19 (10.60)	40.68 (9.18)	41.41 (10.59)	2.13 (−0.28 to 4.55; 0.08)			1.00 (−1.67 to 3.68; 0.46)		
26	43.54 (10.82)	43.50 (10.64)	46.02 (10.47)	1.62 (−0.79 to 4.03; 0.19)			−0.27 (−2.89 to 2.34; 0.84)		
SF-12 mental health component (crude mean (SD) score on quality of life (0–100); lower score indicates less quality of life; adjusted mean difference measured between groups¶¶)									
12	47.27 (10.21)	50.04 (9.00)	48.25 (8.90)	−**3.70 (−6.49 to 0.92; 0.01**)			−**3.23 (−6.33 to −0.14; 0.04**)		
26	47.54 (10.47)	49.30 (9.19)	48.97 (8.90)	−**3.00 (−5.71 to 0.29; 0.03**)			−2.99 (−5.96 to −0.03; 0.05)		

All analyses are adjusted for age, BMI, sex and activity level according to SQUASH.

*Sham group is reference group

†Usual care group is reference group;

‡Analysis is also adjusted for educational level, bilateralism of pain, other musculoskeletal pain, self-reported illness in last 12 months, physical component of the SF 12 at baseline, mental component of the SF12 at baseline, pain score during activity and the disability subscore of the FFI at baseline.

§Analysis is also adjusted for duration of complaints, bilateralism of pain, the physical component of the SF12 at baseline, self-reported illness in last 12 months, other musculoskeletal pain, pain score at rest at baseline and the disability subscore of the FFI at baseline.

¶Analysis is also adjusted for duration of complaints, bilateralism of pain, educational level, physical component of the SF12 at baseline, pain score at rest at baseline and the disability subscore of the FFI at baseline.

**Analysis is also adjusted for duration of complaints, bilateralism of pain, educational level, physical component of the SF12 at baseline, mental component of the SF12 at baseline, pain score at rest at baseline, pain score during activity at baseline, self-reported illness in last 12 months, other musculoskeletal complaints and the disability subscore of the FFI at baseline.

††Analysis is also adjusted for duration of complaints, bilateralism of pain, educational level, physical component of SF12 at baseline, mental component of SF12 at baseline, pain score at rest at baseline, pain score during activity at baseline, self-reported illness in last 12 months and other musculoskeletal pain.

‡‡Analysis is also adjusted for duration of complaints, bilateralism of pain, educational level, physical component of SF12 at baseline, mental component of SF12 at baseline, pain score at rest at baseline, pain score during activity at baseline, self-reported illness in last 12 months, other musculoskeletal pain and the disability subscore of the FFI at baseline.

§§Analysis is also adjusted for educational level, bilateralism of pain, pain score at rest at baseline, pain score during activity at baseline, self-reported illness in last 12 months and the disability subscore of the FFI at baseline.

¶¶Analysis is also adjusted for duration of pain, bilateralism of pain, the physical component of the SF12 at baseline, pain score during activity at baseline, self-reported illness in last 12 months and other musculoskeletal pain.

BMI, body mass index; FFI, Foot Function Index; MD, mean difference; NNT, number needed to treat; RR, relative risk; SF12, The 12-Item Short Form Health Survey; SQUASH, Short Questionnaire to Assess Health-enhancing Physical Activity.

### Co-interventions and compliance

Reported co-interventions up to 12 weeks follow-up are presented in table 3. Patients allocated to usual care reported more visits to the GP (46.2% vs 27.3%, respectively), more use of heel cups or other biomechanical interventions (41.0% vs 22.7%, respectively) and received a corticosteroid injection more often (15.4% vs 0%, respectively) compared with both insole groups. Newly reported co-interventions at 26 weeks, that were not reported at 12 weeks, are presented in online supplementary file 1. One hundred and eighteen patients that were allocated to an insole intervention completed questions on their compliance and satisfaction at 26 weeks of follow-up. Of these, 68 (57.6%) reported wearing their insoles every day (51.7% in the custom-made insole group vs 63.3% in the sham insole group), 10 patients (8.4%; 5 patients in each group) reported never wearing them and 40 patients (33.6%; 39.6% in the custom-made insole group and 28.3% in the sham insole group) reported wearing their insoles sometimes, with no differences between groups. Blinding appeared to be successful as shown in table 4.

**Table 3:** Co-interventions reported at 12 weeks follow-up

Intervention	12 weeks			
	Custom-made insole (n, %) (n=66)*	Sham insole (n, %) (n=68)*	Usual care (n, %) (n=39)*	P value
Consulted with healthcare practitioner†				
General practitioner	18 (27.3)	16 (23.5)	18 (46.2)	0.04
Specialist	1 (1.5)	3 (4.4)	–	0.30
Physiotherapist	6 (9.1)	7 (10.3)	5 (12.8)	0.83
Other healthcare provider (ex. acupuncturist)	1 (1.5)	2 (1.5)	–	0.53
Interventions				
Pain medication	18 (27.3)	21 (30.9)	15 (38.5)	0.49
Exercises	42 (63.6)	45 (66.2)	21 (53.8)	0.43
Shockwave	–	2 (2.9)	2 (5.1)	0.22
Dry needling	1 (1.5)	2 (2.9)	–	0.53
Massage/manipulation	4 (6.1)	–	2 (5.1)	0.13
Other biomechanical interventions‡	15 (22.7)	9 (13.2)	16 (41.0)	<0.001
Shoe advice	11 (16.7)	7 (10.3)	7 (17.9)	0.44
Corticosteroid injection	–	–	6 (15.4)	<0.001

*The N is given for the number of patients that has completed the part of the questionnaire regarding co-interventions. Values are numbers (percentages) unless stated otherwise

†Contacts do not include those as part of trial interventions or those mentioned as cross-over in the flowchart.

‡Other biomechanical interventions included prefabricated insoles, heel cups, night splints or Strasbourg socks, supportive stockings or tape.

**Table 4:** Answers to question regarding blinding, asked at 26 weeks follow-up to patients allocated to either sham or insole

Answer to question: ‘Which intervention do you think you received?’ Asked at 26 weeks follow-up to patients that were allocated to either sham or insole	Allocated to custom-made insole (n, %) (n=70)	Allocated to sham insole (n, %) (n=69)
Sham	12 (17.1%)	13 (18.8%)
Insole	20 (28.6%)	21 (30.4%)
Don’t know	26 (37.1%)	26 (37.7%)
No answer	12 (17.1%)	9 (13.0%)

For 131 participants allocated to an insole, the podiatrist reported the agreement with the referral. In 96.2% of these participants they agreed with the indication for insoles, in the other five cases the podiatrist indicated that normally they would have chosen another type of treatment. Negative effects of the insoles in our study included discomfort when wearing the insoles (reported by 31 participants: 8 in sham group and 23 in insole group) and an increase in pain when wearing the insole (reported by 22 participants; 7 in sham group and 15 in insole group).

### Predefined subgroup analyses

Predefined subgroup analyses were performed on participants with a duration of complaints <12 months at baseline (n=160), participants in which the podiatrist agreed with the referral (n=180) and patients with a high or intermediate activity (in tertiles) based on the SQUASH (n=124). These results are presented in online supplemental file 2. Among participants with short duration of complaints, the differences in pain during activity between the insole and the usual care group were no longer significant, but the size and direction of effect were similar.

10.1136/bjsports-2019-101409.supp1 Supplementary data

### Patient and public involvement

No patients were involved in the planning and development of this study. The results will be disseminated directly to all participants via email.

## Discussion

This study found no differences between a custom-made insole and a sham insole on pain, function, recovery or quality of life in patients with plantar heel pain. Patients with plantar heel pain treated with GP-led usual care experienced less pain, better function and greater improvement compared with patients allocated to the custom-made insole intervention. However, the differences found were small and did not reach clinical importance except for foot function.^21 23^ In the subgroup analysis for participants with a relatively short duration of complaints, the differences in pain during activity between the custom-made insole and the usual care group were no longer significant. The direction of effect was however comparable.

### Comparison with existing literature

To our knowledge, this is the first randomised trial that compares the effectiveness between two commonly used treatment approaches: GP-led usual care versus referral to a podiatrist for custom-made insoles. Our results show no superiority of a custom-made insole over a sham insole and over usual care. Two systematic reviews found conflicting evidence for the effectiveness of custom-made insoles.^12 13^ A randomised controlled trial (RCT) published after these reviews, found a significant effect on first step pain in favour of custom-made insoles in new shoes versus sham insole in the patients regular shoes.^24^ The most recent systematic review comparing different treatment options for plantar heel pain found that none of the commonly used treatments (including orthoses) were better than the other and that data on long-term effects was lacking.^25^ So the findings of our study align with the most recent systematic review synthesis, including 20 RCTs on efficacy of foot orthoses. No previous randomised trials included a usual care treatment strategy in primary care.

### Strengths and limitations

This study adds to literature, since it provides a comparison of custom-made insoles and usual care of the GP in patients with plantar heel pain. Usual care in our study consisted of the care that the GP would normally provide for these patients and all participants additionally received an information booklet with exercises. Participants in the usual care group reported more visits to the GP and more use of freely available biomechanical interventions and corticosteroid injection as co-interventions at 12 weeks. Biomechanical interventions such as heel cups, and corticosteroid injections are common interventions by GPs.^8^ Since the use of biomechanical interventions was questioned by a single yes-no question, we do not have specific information on the time that these interventions were used. It is possible that this affected the results. The use of pain medication (apart from corticosteroid injection) was comparable between groups throughout the entire study. However, the GPs may have applied more interventions, that is, corticosteroid injections, pain medication and other biomechanical interventions to patients in the usual care group in this trial than what is reflective for usual care (less corticosteroid injections (1.4%) and less pain medication (19.9%)).^7^ This may have influenced the treatment effects of usual care in this trial. An additional subgroup analysis excluding the six patients who had received a corticosteroid injection found a comparable MD as the main analysis. This subgroup analysis may be biassed since patients who received a corticosteroid injection seemed to have more severe complaints at baseline. The GP-led usual care group had access to more co-interventions, which might have enhanced the treatment effects in this group. The co-interventions were comparable between the custom made insole group and the sham insole group.

Information on adherence to the podiatry-led interventions was only collected at 26 weeks of follow-up and may be influenced by recall bias.

The sham insoles may have mechanical effects on the foot, and blinding might be an issue.^26 27^ The sham intervention used in this study was especially designed by podiatrists to minimise any mechanical effect, but this cannot be ruled out. Neither were any biomechanical tests applied to test these possible effects. We did observe successful blinding of the patients (table 4).

No patients were included by participating sports physicians. The study results might therefore not be generalisable to a population with a high activity level. Moreover, the inclusion of patients was based on the clinical diagnosis by the GP and we did not include the specific criteria of pain at palpation of the medial tubercle of the calcaneus, as in prior RCTs of plantar heel pain.^28 29^

One of our primary outcomes was pain at rest. We did not find any differences between the groups for this outcome at any time point, in contrast to pain during activity and first step pain. Scores for pain at rest were relatively low at baseline, which might reflect that walking after a period of inactivity is most burdensome for patients with plantar heel pain. Pain at rest may not be an appropriate outcome for patients with plantar heel pain.

### Implications for research and practice

Since this is the first RCT in patients with plantar heel pain using usual care by the GP as a treatment arm, more studies on plantar heel pain including a comparison of usual care (GP-led or other) or watchful waiting are needed. When communicating to patients and clinicians about usual care, it is important to explain that this is not the same as doing nothing. Exercise-therapy and general advice should be included in these approaches. Taking the results of this study into account, GPs can apply a usual care approach, including different types of interventions, when treating plantar heel pain. Since custom-made insoles do not have an additional beneficial effect over sham insoles and GP-led usual care, healthcare providers should be reserved in prescribing custom-made insoles to patients with plantar heel pain.

**Summary box**

**What are the findings?**

- In patients with plantar heel pain in general practice, treatment with custom-made insoles does not have additional value compared with the usual care provided by the general practitioner.
- No differences were found between custom-made insole and sham insole treated patients with plantar heel pain.

**How might it impact on clinical practice in the future?**

- Healthcare providers such as GP’s and podiatrists should be reserved in prescribing custom-made insoles to patients with plantar heel pain.
