# Supplementary material for: Custom insoles versus sham and GP-led usual care in patients with plantar heel pain: results of the STAP-study - a randomised controlled trial
Source: Br J Sports Med. 2020 Sep 2;55(5):272–8. doi: 10.1136/bjsports-2019-101409 (PMC7907578; doi:10.1136/bjsports-2019-101409)
Supplement: Supplementary data [file bjsports-2019-101409supp001.pdf]

	Duration of complaints of < 12 months at baseline (N=160)		Participants in which the podiatrist agreed with the referral (N=180)		High or intermediate score on the SQUASH activity score at baseline (in tertiles) (N=124)	
No of weeks after start of intervention	Insole vs sham* **MD (95% CI; P)**	Insole vs Usual care** **MD (95% CI; P)**	Insole vs sham* **MD (95% CI; P)**	Insole vs Usual care** **MD (95% CI; P)**	Insole vs sham* **MD (95% CI; P)**	Insole vs Usual care** **MD (95% CI; P)**
Pain at rest score on pain numerical rating scale (0-10); lower score indicates less pain; adjusted mean difference measured between groups[1])						
6	-0.41 (-1.15 to 0.33; 0.28)	-0.34 (-1.25 to 0.56; 0.45)	-0.35 (-1.06 to 0.37; 0.34)	-0.27 (-1.10 to 0.57; 0.53)	0.06 (-0.75 to 0.88; 0.88)	-0.25 (-1.31 to 0.82; 0.65)
12	-0.28 (-1.01 to 0.44; 0.44)	-0.16 (-1.03 to 0.71; 0.71)	-0.28 (-0.97 to 0.42; 0.44)	-0.12 (-0.92 to 0.66; 0.77)	0.06 (-0.74 to 0.86; 0.88)	-0.16 (-1.17 to 0.86; 0.76)
26	-0.24 (-0.95 to 0.46; 0.50)	-0.19 (-1.06 to 0.68; 0.67)	-0.26 (-0.93 to 0.40; 0.44)	-0.15 (-0.94 to 0.64; 0.71)	0.11 (-0.66 to 0.88; 0.78)	-0.18 (-1.17 to 0.82; 0.73)
Pain during activity score on pain numerical rating scale (0-10); lower score indicates less pain; adjusted mean difference measured between groups[2])						
6	-0.15 (-0.77 to 0.46; 0.62)	0.70 (-0.10 to 1.5;0.09)	-0.02 (-0.58 to 0.54; 0.95)	1.02 (0.31 to 1.74; 0.01)	-0.30 (-0.94 to 0.33; 0.35)	1.05 (0.18 to 1.93; 0.02)
12	-0.07 (-0.67 to 0.53; 0.81)	0.70 (-0.09 to 1.50;0.08)	0.00 (-0.55 to 0.55; 0.99)	1.01 (0.30 to 1.72; 0.01)	-0.27 (-0.91 to 0.36; 0.39)	1.03 (0.17 to 1.90; 0.02)
26	0.10 (-0.49 to 0.69; 0.73)	0.69 (-0.11 to 1.48;0.09)	0.12 (-0.43 to 0.66; 0.68)	0.98 (0.28 to 1.69; 0.01)	-0.08 (-0.73 to 0.57; 0.82)	1.01 (0.15 to 1.87; 0.02)

*Sham group is reference group ** Usual care group is reference group; MD= Mean Difference, CI= Confidence interval, FFI= Foot Function Index, SF12= The 12-Item Short Form Health Survey; All analyses are adjusted for age, BMI, gender and activity level according to SQUASH [1] Analysis is also adjusted for educational level, bilateralism of pain, other musculoskeletal pain, self-reported illness in last 12 months, physical component of the SF 12 at baseline, mental component of the SF 12 at baseline, pain score during activity and the disability sub score of the FFI at baseline. [2] Analysis is also adjusted for duration of complaints, bilateralism of pain, the physical component of the SF12 at baseline, Self-reported illness in last 12 months, other musculoskeletal pain, pain score at rest at baseline and the disability sub score of the FFI at baseline.

Supplementary file 2. Results of the pre-defined subgroup analysis on the primary outcomes pain at rest and pain during activity
